# Central Dental Association of Northern New Jersey

**Published:** 1884-03

**Authors:** 


### CENTRAL DENTAL ASSOCIATION OF NORTHERN NEW JERSEY.

The regular monthly meeting of this Society was held at the office of Dr. F. C. Barlow, No. 646 Jersey Avenue, Jersey City, N. J., December 27, 1883.

#### INCIDENTS OF OFFICE PRACTICE.

*Dr. Watkins.*—I have here a tooth extracted the other day, which was filled several years ago with gold. The pulp was dead, the root was not filled, and of course there was subsequent disturbance. It came out very easily, was bathed in pus, and on examination I found that half the root still remained in the mouth. The root had been split, and to my apprehension this was done by the pressure of gas within the pulp chamber.

*Dr. Atkinson.*—There is no other explanation that will contradict that as far as I can see. I have been acquainted with many instances, two of which were loud enough to be heard at quite a distance.

My father and my sister-in-law afford two examples of explosion sufficiently loud to be heard. I remember we could not believe a tooth had exploded, but it gave relief. I do not think we are able to tell what it is that makes the change in the molecules, and generates the gas so suddenly that it becomes such a powerful explosive.

*Dr. Watkins.*—I questioned this gentleman regarding the case. He could not remember experiencing any explosion in the tooth.

*Dr. Luckey.*—On Monday a gentleman called at my office whose teeth, with the exception of the first two molars in the upper jaw, were as perfect as any I have ever seen. I noticed along the roots of the right and left upper molar there was absorption of the soft tissue. He wanted an explanation, and desired to know if it would be a serious matter, and whether he would have to lose the teeth. I looked in vain for foreign bodies; there was no irritation, no tartar to be found, and I could attribute it to no discoverable cause. I present the case now to learn if any gentleman present can account for it.

*Dr. Atkinson.*—We often see this condition, and a great many practices are charged with it ; tobacco chewing, smoking, and the tooth brush have been accused. My impression is that these teeth have less longevity than any others, and run life out much sooner by reason of want of nutrition.

*Dr. Luckey.*—Is there any successful treatment for this case ?

*Dr. Atkinson.*—Yes; you could make a pocket, so as to leave a place or a space to allow for the growth of new tissue, by means of a properly constructed plate. I have a case that is very interesting; one that I have treated since last August. The tooth was knocked loose over thirty years ago, and when it came to me there was not enough connection between the dental membrane and the tooth to keep it upright. He could play it back and forth with the tongue very readily. I tied it in place, and the tissue is now so far reproduced as to leave only space enough for inserting a small syringe point, with no indications of pus at all, while previously it had been discharging from the nostril. The ligature can soon be removed, and it be left to nature.

*Dr. Luckey.*—I had a case where the patient suffered from soreness in the right central, and yet there did not seem to be any swelling at the apex of the roots, but I saw from the color that the pulp had been dead for some time. I drilled into the pulp chamber, and there was a great discharge of pus and bloody serum. We could not stop the discharge, which seemed to come from the nose, but he said he had experienced relief.

*Dr. Atkinson.*—Certainly he had catarrh from the nose, and that was where the point of relief was. You can stop the discharge with chloroform, or what is better, carbolic acid on the end of a stick, and just where it is beginning to turn white cut through and, after cleansing, fill the root, and it will be cured.

*Dr. Palmer.*—I will relate an incident that happened to one of our profession in Pittsburgh, Pa. A short time ago a gentleman called to have his teeth put in order before going to Cuba. Examination showed that they were all very loose. The gentleman wanted them filled. The dentist cleaned them thoroughly, going well under the gums, and then applied sulphate of copper. He then dismissed the gentleman, who was very indignant that the dentist should refuse to fill the teeth. The doctor said they were entirely too loose, and that time must be allowed to heal them. The gentleman wanted to know if that was the way he always treated his patients, and went away much incensed. About a year after, the same gentleman entered my friend's office with the remark, "Will you fill my teeth now?" Examination showed that the teeth were as firm as ever—one *thorough* cleansing and application had accomplished the work.

*Dr. Atkinson.*—If you cannot cure with one application you can with fifty—that is, if you will persist in the use of iodoform.

*Dr. Richards.*—A lady came into my office this afternoon. I noticed in taking an impression of her mouth that one tooth was a bright, claret color. I have often noticed it before, but never knew the cause of it.

*Dr. Atkinson.*—It is infiltration of the dentine. The coloring matter has four or five names—hemoglobine, hematine, etc. It is the red coloring matter of the blood.

*Dr. Watkins.*—Since the meeting at my house I have had several enquiries made in regard to extracting and replacing teeth. The case which I showed that night has been perfectly successful. The next case I wish to speak of is that of a young lady about twenty-three years of age. The pulp of a second bicuspid was considerably congested, and she insisted upon having it out. I could not persuade her to have the tooth treated and filled, but after extracting it I insisted on putting it back again. I first trimmed the root off about one-sixteenth of an inch, and syringed the socket with a weak solution of carbolic acid. I removed the nerve, filled the canal with gutta-percha, put a little iodoform on the root, and tied the tooth in with silk floss, and in six days she said there was not a particle of soreness. I have seen her frequently since, and it seems to give her no trouble at all, and she considers it just as good as any tooth in her mouth. The next case is that of a young man about nineteen years of age. His first left superior bicuspid I treated in the same way, except that I used nothing to syringe the socket out, and at the end of seven days he could eat with it, and it seemed to be entirely well. It seems to me that in many cases where the patient lives at a distance and would have to come several times for treatment, it would be most judicious to extract, fill and replant.

*Dr. Ayers.*—A young lady once came to me, for whom a physician had extracted the wrong tooth. I proposed to replant it, but the tooth was lost, and only found after a prolonged search, in the coal scuttle. I washed the cavity out, replaced the tooth, and now, after a year and a half, it remains in good condition.

*Dr. Merritt.*—One day last week a lady came into my office, for whom a tooth had been extracted sixteen years ago, and put back, and it is apparently in as good condition as ever, and is doing full duty.

*Dr. Barlow.*—Related a number of instances of successful replantation.

*Dr. Palmer.*—A young lady came into my office two weeks ago. I think she had been a patient of mine off and on for several years, but this was the first time I had seen her in four years. I examined her teeth, and among other things found one central incisor dead. On questioning her I learned that a blow or fall had knocked out the tooth. Their family physician was called, and arriving some hours after, he had replaced the tooth. It was sore only a few days, and had never given trouble. This was three years before. The physician told her it would never trouble her. It was *very* dark. I suggested opening, treating and bleaching it. Objections were raised because the M. D. had said it was all right. After some persuasion I gained my point—opened, treated and closed the tooth successfully. Immediately upon opening there appeared enough pus at the orifice to convince them that I was right. In this case I wonder if Dr. Watkins would prefer to re-extract, cleanse, and then replace? I preferred to treat it in the mouth. And I want to call attention to the fact that this physician did not know more than any one else, when he said the tooth would never give any trouble.

*Dr. Levy.*—I have heard about replacing the same teeth. I would like to know whether any have replaced other teeth.

*Dr. Luckey.*—There is no doubt that the replantation of teeth is very successful in many instances. There is one great objection to Dr. Watkins' plan; you never know in what condition the roots are by looking in the mouth. You cannot see exostosis, and when such teeth are once out there is no chance of getting them back.

*Dr. Watkins.*—In most cases of exostosis the only radical cure is extraction, and replantation would not of course be desired.

*Dr. Palmer.*—If a tooth were firm in its socket I should be inclined to treat it in the mouth rather than to extract it; but if it were elongated and loose, and the patient lived at some distance, I can see no objection to Dr. Watkins' practice. A leading physician of this State once came to me, insisting upon the extraction of a troublesome tooth. Examination showed a small cavity in the mesial surface of a left superior lateral incisor, extending into the pulp chamber, with an abscess discharging through the tooth. I advised treatment and filling. He was positive that there was no cure; that such a thing was contrary to physiological law; but he finally yielded to my persistence, though, as he said, against his judgment. To-day that tooth is doing good service, and the physician is at last convinced that dentists may know more about some portions of the body than even a successful physician.

On motion the society adjourned.

